# Precise control over shape and size of iron oxide nanocrystals suitable for assembly into ordered particle arrays

**DOI:** 10.1088/1468-6996/15/5/055010

**Published:** 2014-10-31

**Authors:** Erik Wetterskog, Michael Agthe, Arnaud Mayence, Jekabs Grins, Dong Wang, Subhasis Rana, Anwar Ahniyaz, German Salazar-Alvarez, Lennart Bergström

**Affiliations:** 1 Department of Engineering Sciences, Ångström Laboratory, Uppsala University, Sweden; 2 Department of Materials and Environmental Chemistry, Arrhenius Laboratory, Stockholm University, Sweden; 3 Department of Chemical Engineering, Northeast Dianli University, People’s Republic of China; 4 Variable Energy Cyclotron Centre, 1/AF, Bidhan Nagar, Kolkata, India; 5 SP Technical Research Institute of Sweden, Chemistry, Materials and Surfaces, Stockholm, Sweden

**Keywords:** iron oxide, nanoparticles, synthesis, assembly, superlattice, mesocrystal

## Abstract

Here we demonstrate how monodisperse iron oxide nanocubes and nanospheres with average sizes between 5 and 27 nm can be synthesized by thermal decomposition. The relative importance of the purity of the reactants, the ratio of oleic acid and sodium oleate, the maximum temperature, and the rate of temperature increase, on robust and reproducible size and shape-selective iron oxide nanoparticle synthesis are identified and discussed. The synthesis conditions that generate highly monodisperse iron oxide nanocubes suitable for producing large ordered arrays, or mesocrystals are described in detail.

## 1. Introduction

The iron oxides are among the most abundant and important minerals on earth. Early processing of iron oxides enabled man to craft paints with different colors, and there is evidence that yellow goethite (*α*-FeOOH) was transformed by heat to produce red hematite (*α*-Fe_2_O_3_) in Troubat (French Pyrenees) over 10 000 years ago [1]. Today, iron oxides are used for a number of applications including: catalysis, magnetic storage, ferrofluids and pigments to name a few [2]. Particularly, many potential uses for nanoscale iron oxides in medicine are being extensively researched [3] e.g. for hyperthermia treatments [4], bio-sensing [5, 6], lab-on-a-chip diagnostics [7], and as contrast agents for magnetic resonance imaging (MRI) [8].

The large interest in nanoscale iron oxides has over the last two decades stimulated a development of methods that can yield particles with well-defined structure, chemical composition, size distribution, shape, and magnetic properties (e.g. magnetic moment and anisotropy) [9–17]. Moreover, the narrow size and shape dispersion attainable by contemporary methods have enabled the fabrication of highly ordered particle superstructures, a topic of both fundamental and technological importance [18].

Modern wet-chemical nanoparticle synthesis is typically performed in non-polar solvents and generally involves the decomposition or reduction of a metal-organic complex e.g. M^*n*+^ (C_6_H_5_N(NO)O^−^)_*n*_ [19], M(CO)_5_ [20], M^*n*+^(acac^−^)_*n*_ [21], or Fe[N(SiMe_3_)_2_]_2_ [22] in the presence of a surfactant. Colloidal synthesis routes provide much greater control over the size distribution of the products compared to e.g. ball milling and aqueous co-precipitation routes [12]. The metal oleate thermal decomposition route, pioneered by Hyeon and coworkers [23], is perhaps the most widely used iron oxide nanoparticle synthesis route today because of its simplicity, scalability and control over the reaction products. Following their initial synthesis of spherical ferrite particles [23] the iron oleate synthesis method was further developed to yield cubic [24–27], and octahedral iron oxide nanoparticles [28] and also, at least in partial yield, other shapes, e.g. trigonal bipyramids [24].

Despite the extensive efforts and large number of papers published on the iron oleate synthesis of iron oxide nanoparticles [17, 24, 29, 30], there is a lack of a comprehensive account on how to simultaneously control the particle size and shape while confining the particle size distribution to limits that are acceptable for formation of mesostructures with long-range order, i.e. nanoparticle superlattices and mesocrystals. Here, we give a comprehensive account on the shape- and size- selective synthesis of highly monodisperse iron oxide nanoparticles that are suitable as building blocks for self-assembly into large, ordered nanoparticle arrays. We elaborate on the important parameters for robust and reproducible nanoparticle synthesis. We discuss effects relating to the purity of the reagents and demonstrate that the size and shape selectivity is partially lost when using low-grade sodium oleate in the synthesis of the iron(III) oleate precursor. Finally, the separation of the particles from the synthesis solution and their assembly into highly ordered mesocrystals is described in detail.

## 2. Experimental

### 2.1. Synthesis of iron oxide nanocrystals

All chemicals were purchased from Sigma-Aldrich or Tokyo chemical industry (TCI) and used as received: iron(III) chloride hexahydrate (97%, Sigma-Aldrich), 1-octadecene (Sigma-Aldrich, 90%), 1-hexadecene (Sigma-Aldrich, 92%) and eicosane (Sigma-Aldrich, 99%). The surfactants (oleic acid, sodium oleate) used in the various nanoparticle syntheses presented herein are subdivided by purity: Reagent group A—oleic acid (99%, TCI), sodium oleate (97%, TCI). Reagent group B—oleic acid (90%, Sigma-Aldrich), sodium oleate (82%, Riedel-de-Haen).

The preparation of the iron oleate precursor follows previous descriptions [23, 27, 30]. In brief, iron(III) chloride (40 mmol) and sodium oleate (40 mmol) are dissolved in a mixture of 60 mL MilliQ water, 60 mL ethanol and 140 mL *n*-hexane in a round bottom flask. The solution is stirred until the reagents have been completely dissolved, after which the solution is refluxed for 4 h. The solution is cooled down and allowed to phase separate; the brown hexane phase is separated from the clear H_2_O/ethanol phase and washed three times with 30 mL MilliQ water. The hexane phase is then transferred into a round-bottom flask, and the solvent is removed using a rotary evaporator, yielding a red-brown viscous product. For practical reasons, the iron oleate precursor was subdivided into smaller portions. In a typical procedure, the iron oleate precursor is dissolved into 50–100 mL of 1-octadecene or 1-hexadecene. The solution is then vacuum dried on a Schlenk line at a pressure <1 mbar at a temperature of 90 °C under magnetic stirring and maintained at that temperature until the bubbling ceases, typically within 1 h.

Iron oxide nanoparticles were synthesized by thermal decomposition of the dissolved precursor in a high-boiling solvent in the presence of oleic acid or a mixture of oleic acid and sodium oleate. The maximum reflux temperature was adjusted by using mixtures of e.g. 1-hexadecene, 1-octadecene and/or eicosane. A typical synthesis is performed in a 250 mL round bottom flask using 50 mL solvent and 10 mmol of iron oleate. For the synthesis of spherical particles, oleic acid is added (1.43–10 mmol, see table 1) whereas for the synthesis of cubic nanoparticles, a mixture of oleic acid (1.43–5 mmol) and sodium oleate (0.715–5 mmol) is added.

**Table 1.** Overview of the synthesis parameters and characteristics of iron oxide nanocubes and nanospheres (*n*_iron oleate_ = 10 mmol).

Name	Reflux temperature (°C)	Heating rate (°C min^−1^)	Reflux time (min)	Oleic acid (mmol)	Sodium oleate (mmol)	Shape	Edge length/diameter and standard dev. (nm)
Reagent group A: 99% oleic acid, 97% sodium oleate						
C094	315	3.0	30	2.145	0.715	Cubes	9.4 ± 0.4(4%)
C096	315	3.0	30	1.43	1.43	Cubes	9.6 ± 0.4 (4%)
C126	310	3.0	30	1.43	1.43	Cubes	12.6 ± 0.8^a^ (6%)
C136	319^b^ (315)	3.0	37	1.43	1.43	Cubes	13.6 ± 0.8 (6%)
C174	325	3.0	30	2.145	0.715	Cubes	17.4 ± 1.2 (7%)
C187	327	3.0	30	2.5	2.5	Cubes	18.7 ± 1.5 (8%)
C230	350	3.3	30	5	5	Cubes	23.0 ± 2.6 (11%)
S050	325	3.0	30	5	0	Spheres	5.3 ± 0.4^c^ (8%)
S157	327	3.0	30	5	0	Spheres	15.7 ± 1.7 (11%)
S270	350	3.3	30	10	0	Spheres	27.0 ± 2.0 (8%)
Reagent group B: 90% oleic acid, 82% sodium oleate						
TC086	320	2.6	30	2.86	0	Cubes	8.6 ± 0.5 (6%)
S091	320	2.6	30	2.86	0	Spheres	9.1 ± 0.6 (7%)
PD820	320	2.2	30	2.86	0	Cubes	8–20^d^

a Deviating from size-trend.

b Superheated (no magnetic stirring).

c Argon bubbling.

d Polydisperse.

The temperature of the synthesis solution is increased to reflux at a heating rate of 2.2–3.3 °C min^−1^ under a blanket of inert gas and held at the reflux temperature for a set time, typically 30 min. The heating rate and holding times are adjusted using a temperature controller (Julabo LC6). Note that the addition of iron oleate results in a slight elevation of the reflux temperature relative to that of the pure solvent(s). Also, if not stirred e.g. magnetically, the solution can superheat. A common problem during thermal decomposition synthesis is the formation of volatile decomposition products that condense in the reflux condenser and drop back into the reaction vessel where they may cause violent splashing and temperature fluctuations. This problem was completely alleviated by attaching a Dean–Stark condenser to the synthesis apparatus. If needed, the flow of inert gas can be momentarily increased in order to drive the vapor to the Dean–Stark condenser. Finally, the solution is allowed to cool to room temperature under a constant flow of inert gas.

### 2.2. Purification and workup of iron oxide nanoparticles and preparation of dispersions suitable for self-assembly

The purification and workup of the nanoparticles in the synthesis dispersion (mother liquor) consist of several important steps. The mother liquor is first shaken with ethanol in a large Erlenmeyer flask resulting in that the majority (≈75%) of the synthesis solvent transfers to the resulting opaque ethanol phase. The mixed solvents are allowed to phase separate and the ethanol phase is decanted (residual ethanol can be removed using a Pasteur pipette). The remaining non-polar synthesis dispersion (≈10–15 mL) is then diluted with a small amount of toluene (1–2 mL) and shaken vigorously for ≈1 h and then mixed with three parts of ethanol and shaken again. The solution is allowed to phase separate and the ethanol phase is decanted. The separation of the dispersion phase containing the iron oxide nanoparticles can be also performed using a strong magnetic field gradient or by centrifugation. We have however found that centrifugation and magnet assisted workup may lead to the formation of aggregates that may be difficult or impossible to break up. In the case of particles which are 8–14 nm in size, 4–5 of the washing cycles (dilution by toluene and ethanol) is typically sufficient to yield a product with 40–50 wt% solids content (determined by thermogravimetric analysis in air at 800 °C). Following the final decantation of ethanol, the nanoparticle dispersion is diluted with a few mL of toluene, shaken for 1–2 h after which the toluene is slowly evaporated under a gentle vacuum at 40 °C. The resulting highly viscous concentrated nanoparticle stock dispersion can be used to prepare dilute dispersions in toluene. If stored under a protective atmosphere and at room temperature we approximate the shelf-life of the stock dispersion to ≈6–12 months. After this, we have found that the quality of the self-assembled arrays derived from it starts to deteriorate. In such cases, the stock dispersion can be replenished by addition of fresh oleic acid during the dilution (see section 3). In the case of large particles (<20 nm), the steric repulsion provided by the oleic acid coating is insufficient to prevent agglomeration and yields a solid like powder following the final evaporation step. Toluene dispersions with a concentration of 2–8 mg iron oxide/mL are typically used for the preparation of large ordered arrays.

Self-assembled mesocrystals/superlattices were produced by allowing 20 *μ*L of a 2 mg mL^−1^ toluene dispersion to slowly evaporate on a 1 cm^2^ Si wafer that is placed in a compartment, e.g. a Petri dish that minimizes convection and also allows the evaporation rate to be controlled. The evaporation rate can be significantly reduced by applying about 80 *μ*L of toluene along the rim of the compartment. For instance using a Petri dish (⊘ = 2″), extends the time window for mesocrystal formation to several hours.

### 2.3. Characterization

The sizes and shapes of the nanoparticles were determined using transmission electron microscopy (TEM). Dilute toluene dispersions of the iron oxide nanoparticles were distributed over carbon-coated TEM grids and analyzed using either a JEOL 2000FX-II (200 kV, LaB_6_ filament, point resolution 3.1 Å), a JEOL 3010 (300 kV, LaB_6_ filament, point resolution 1.7 Å) or a JEOL 2100 microscope (200 kV, LaB_6_ filament, point resolution 2.5 Å). Size distributions of the nanoparticles were determined by measuring the edge length (or diameter) of 200–300 particles of each sample. The measured cubic particles had the [001] zone axis parallel to the electron beam; cubes that were clearly misaligned (tilted) were disregarded. The image magnification was calibrated against the lattice fringe spacing *d*_220_ ≈ 2.968 Å of Fe_3_O_4_.

The self-assembled nanoparticle arrays were imaged using a JEOL-7000 F scanning electron microscope (SEM) at an accelerating voltage of 20 kV. Images were filtered using a Fourier mask in the Gatan Digital Micrograph program to reduce noise and increase image clarity. Reflected light microscopy images of the nanoparticle arrays were acquired on a Nikon FN-1 microscope equipped with a 50× objective. Mesocrystal surfaces were imaged using a Veeco Multimode atomic force microscope (AFM) operated in tapping mode with a *μ*masch probe (NSC18, tip radius < 10 nm, *f*_*r*_ = 75 kHz, *k* = 3.5 N m^−1^).

Powder x-ray diffractograms were acquired at the I711 beamline of the MAX-Lab synchrotron in Lund, Sweden. Dispersions of the nanoparticles were evaporated onto thin kapton films. The background was accounted for by subtracting the scattering of a kapton reference sample. The wavelength of the incident beam was 1.001 Å and scattering patterns were recorded on a Titan 2D detector (2048 × 2048 pixels, pixel size 60 *μ*m). The patterns were integrated to 1D profiles with the Fit2D software using LaB_6_ powder as calibrant. Rietveld refinements were performed using the Fullprof software [31]. A Lorentzian size broadening for reflections with *h* = 2*n* + 1 and *k* = 2*m* + 1 (size model number 9) was used to improve the fit between the observed and calculated patterns. A final correction of the refined unit cell parameters was performed using a Rietveld fit of the LaB_6_ standard using *a* = 4.15 691(8) Å [32]. The full width at half maximum (FWHM) was determined for selected reflections by removing the background and fitting individual reflections using the STOE WIN XPOW program (v. 2.24) and a pseudo-Voigt profile function.

## 3. Results and discussion

Iron oxide nanoparticles have been synthesized by thermal decomposition of an iron oleate precursor and the particle size and shape was controlled by the reflux temperature, the excess surfactant concentration, and the reflux time.

**Figure 1.**
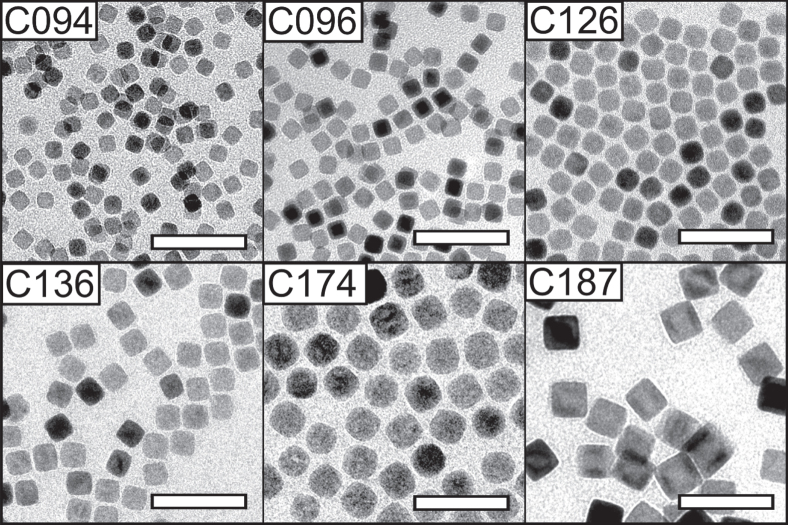
Cubic iron oxide nanoparticles. Transmission electron microscopy (TEM) images of cubic iron oxide nanocubes of different sizes (9–20 nm) synthesized by thermal decomposition of iron(III) oleate in the *presence* of sodium oleate (reagent group A: see table 1). Scale bars: 50 nm.

**Figure 2.**
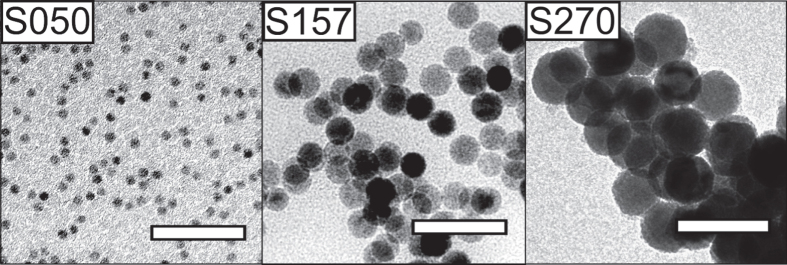
Spherical iron oxide nanoparticles. TEM images of iron oxide nanoparticles of different sizes (5–27 nm) synthesized by thermal decomposition of iron(III) oleate in the absence of sodium oleate (reagent group A, see table 1). Synthesis of the smallest particles (S050) was achieved by bubbling a stream of dry argon through the synthesis solution for the entire duration of the experiment. Scale bars: 50 nm.

Table 1 summarizes how a number of synthesis parameters influence the size of the produced iron oxide nanoparticles. Figure 1 shows representative TEM images of synthesized nanocubes and figure 2 shows representative images of the synthesized nanospheres. Tuning the reflux temperature, i.e. by using pure or binary mixtures of: 1-hexadecene (boiling point (bp) = 287 °C), 1-octadecene (bp = 318 °C), and eicosane (bp = 343 °C), and using different amounts of excess surfactant, had a large effect on the size and size distribution and resulted in iron oxide nanoparticle with sizes between 8 and 27 nm. The optimal size range for the iron oleate decomposition method appears to lie around 8–14 nm, where size distributions with a standard deviation (*σ*_std_) of 4–6%, are readily obtained (see table 1). Overall, controlling the reaction temperature is a straightforward way to generate a library of particles with different sizes and narrow size distributions. The predictability is however not absolute and we have observed deviations from the expected trend in singular cases (see sample C126).

In addition, very small particles (S050) can be synthesized by bubbling a stream of inert gas through the solution for the entire duration of the reaction, as initially suggested by Lynch and co-workers [29]. Moreover, we have found that varying the heating rates between 2.6 and 3.3 °C min^−1^ inferred negligible effects on the shape or size of the nanoparticles. Singular experiments with lower heating rates (2.2 °C min^−1^) have resulted in the formation of polydisperse particles (see sample PD820), but no extensive investigation of this effect has been conducted.

Attempts have previously been made to model the influence of the reaction temperature on the size and the size distribution of iron oxide nanoparticles synthesized by the oleate route [17, 23, 29, 30]. The well-studied increase of the particle size with the amount of excess oleic acid has been ascribed to a modification of the surface reaction rate, according to the simulations of van Embden *et al* [33]. Identifying a mechanism that provides a link between the reaction temperature and the average nanoparticle size has been met with limited success. Although it can be simplistically argued that nanoparticle growth should be faster at higher temperatures [17], a complete theoretical model needs to account for the temperature (and time) dependence of at least four different mechanisms: monomer formation, nucleation, growth, and Ostwald ripening. Further complications arise from the sigmoidal time-dependent monomer formation [34], which indicate that the thermal decomposition reaction involve both acceleratory and deceleratory mechanisms [35], similar to e.g. autocatalytic reactions [34].

**Figure 3.**
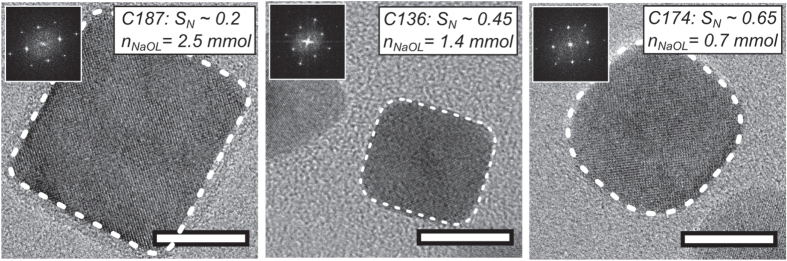
Variation of particle shape with the amount of added sodium oleate. The fast Fourier transform (FFT) pattern of each nanocube is shown as an inset. Dashed lines have been added to highlight the projected contour of the nanocubes. Scale bars: 10 nm.

The nanoparticle shape becomes invariably cubic (see figures 1 and 2) when the decomposition reaction of the iron oleate complex is performed in the presence of a small amount of sodium oleate. Figure 3 shows how the shape of the iron oxide particles depends on the amount of sodium oleate added to the reaction. The shape was quantified using a geometric index, *S*_*N*_, defined as the normalized ratio of the minimum Feret diameter (i.e. minimum caliper diameter) of the particle and the diameter of an inscribed circle with the same area as the particle. A segmented image was used to compute the area (*A*), and the minimum Feret diameter (min_Feret_) of each nanoparticle using the Image J program. Ill-segmented and overlapping particles where manually removed from the image analysis. From the area, the diameter of a circle with an equivalent area is derived, i.e. 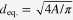. A geometric index 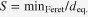 can then be used to estimate the shape of the nanoparticles, taking values between 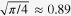 for a square (a projected cube) and 1 for a circle (a projected sphere). At least 50 particles where used to estimate *S*. The *S* parameter can be renormalized to 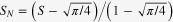, yielding values between *S*_*N*_ = 0 (for a projected cube) and *S*_*N*_ = 1 (for a projected sphere). For the three cube samples shown in figure 3, we observe a reduction of *S*_*N*_ from 0.65 to 0.2 as the amount of added sodium oleate is increased from 0.7 to 2.5 mmol, respectively. Nanoparticles synthesized in the absence of sodium oleate are spherical, with *S*_*N*_ ∼ 1, although the largest nanospheres in this study appear slightly faceted (see sample S270, figure 2) Note that the average particle size is insignificantly affected by the sodium oleate/oleic acid ratio if the total surfactant concentration is kept constant, see samples C094 and C096 in table 1 where the sodium oleate/oleic acid ratio varies from 1/1 to 1/3, respectively.

We speculate that the structure directing effect of sodium oleate is related to the ease of dissociation and the ability of the dissociated surfactants to either suppress the surface growth rates for the {111} facets, or accelerate the growth of the {100} facets. Previous work [24, 28], observed a sharp increase of the conductivity of the synthesis mixture above 220 °C, which was related to surfactant dissociation. The interaction between the dissociated surfactant and the specific crystal facets are expected to be influenced by the difference in surface charge of the {100} and the {111}-planes of Fe_1−*x*_O and Fe_3_O_4_. The {100} planes contain mixed charges, whereas the {111} planes are either positively or negatively charged.

Nanoparticle synthesis using high-purity surfactants (i.e. reagent group A: 97% purity sodium oleate, and 99% purity oleic acid) results invariably in cubic nanoparticles when sodium oleate is added. However, using low-grade (>82%) sodium oleate (reagent group B) for the synthesis of the precursor complex frequently resulted in cubic nanoparticles without any addition of sodium oleate, with a few exceptions (S091).

**Figure 4.**
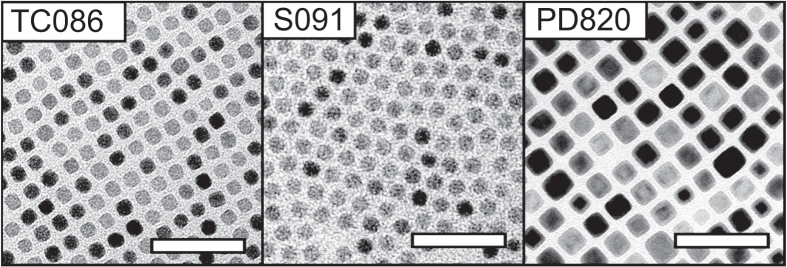
TEM images of iron oxide nanocrystals synthesized by thermal decomposition using low-purity surfactants (reagent group B, see table 1) at a temperature of 325 °C. Scale bars: 50 nm.

We have also found that nanocrystals synthesized using low-grade (82%) sodium oleate (reagent group B, figure 4) are significantly smaller than nanoparticles synthesized at the same temperature from high-grade (97%) sodium oleate (reagent group A). We speculate that compositional differences between the sodium oleates of different purity, there is e.g. a possibility that the low-grade sodium oleate contain fatty acids of varying chain lengths [36, 37], that result in iron-precursors with different decomposition temperatures.

Rietveld refinements of synchrotron powder x-ray diffraction (PXRD) data were performed on nanocubes C096, C126 and C136 after purification and upgrading, see table 2. The criteria of fit between the observed and calculated patterns were high, with *χ* ^2^ values around 40 and structure factor, *R*_F_, values around 5–8%. The discrepancy between the data and calculated patterns can be mainly attributed to anisotropic line broadening effects, and (counter-intuitively) the good counting statistics of the synchrotron data. In order not to underestimate the errors in the obtained unit cell parameters and Fe_1−*x*_O contents, the error values have in table 2 been multiplied by a factor of 10. The smallest particles (9.6 nm) have a Fe_3−*δ*_O_4_ type structure with a lattice parameter slightly smaller than the lattice parameter of bulk magnetite (Fe_3_O_4_, *a*_*0*_ = 8.396 Å) [2], but larger than maghemite (*γ*-Fe_2_O_3_, *a*_*0*_ = 8.348 Å) [38]. The larger particles (C126, C136) with average edge lengths of *l* = 12.6 and 13.6 nm have a core|shell structure with 3 and 5 wt% Fe_1−*x*_O respectively. Moreover, both the C126 and C136 Fe_1−*x*_O|Fe_3−*δ*_O_4_ nanocubes have Fe_3−*δ*_O_4_ lattice parameters which are slightly expanded (≈0.2%) with respect to stoichiometric Fe_3_O_4_, resulting from the epitaxial mismatch between the Fe_3−*δ*_O_4_ shell and the Fe_1−*x*_O core [39].

**Table 2.** Refined powder x-ray diffraction data for the nanocube samples C096, C126 and C136.

			Peak widths at half-max, FWHM (°)
Sample	Fe_3−*δ*_O_4_ lattice parameter (Å)	Fe_1−*x*_O cont. (wt%)	220	400
C096	8.388(2)	—	1.55	0.67
C126	8.416(4)	3(2)	1.57	0.64
C136	8.413(3)	5(2)	1.46	0.68

**Figure 5.**
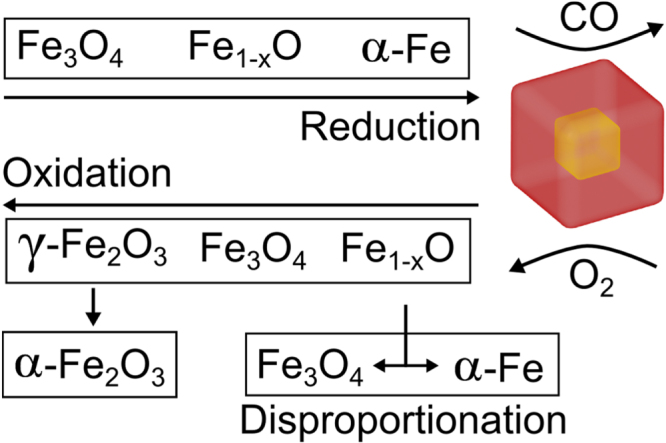
An overview of the chemical transformations in the Fe-O system, that results in the formation of core|shell particles. Reduction of Fe occurs during the synthesis in the presence of reducing agents e.g. CO at high temperatures. Oxidation occurs in post synthesis workup under aerobic conditions. Transformations from metastable phases (Fe_1−*x*_O and *γ*-Fe_2_O_3_) are shown by downward arrows.

The observed core|shell structure is a consequence of the reducing reaction environment. Previous work has shown that thermal decomposition of metal oleates generates several by-products such as CO and CO_2_ gases [34, 40]. At high temperatures, CO(g) reduces Fe^III^ resulting in nanoparticle compositions with varying oxidation states: Fe_3_O_4_, Fe_1−*x*_O and Fe, and in many cases particles with a core|shell structure (see figure 5): Fe_1−*x*_O|Fe_3−*δ*_O_4_ and Fe_1−*x*_O|Fe [25, 40, 41]. Hence, purification and storage of Fe_3_O_4_ and Fe_1−*x*_O nanoparticles in the presence of atmospheric oxygen causes the smaller particles (C096) to oxidize relatively quickly into single-phase particles and eventually to transform completely to a Fe^III^ oxide, i.e. *γ*-Fe_2_O_3_ (see figure 5).

Moreover, peak-profile analysis of the XRD data shows significant anisotropic line broadening, evidenced by a large discrepancy between the FWHM of e.g. the 220 and 400 diffraction lines in the C096, C126 and C136 nanocubes (see table 2). This supports the notion of defects in the cationic sub-lattice of the spinel structure such as anti-phase boundaries (APBs). Previous works have shown that APBs in ferrite particles can have a drastic impact on their magnetic properties inducing e.g. exchange bias, high-field susceptibilities and a lower saturation magnetization compared to the corresponding bulk phases [39, 42].

After the synthesis is completed, the resulting mother liquor contains about ≈1 wt% of solid material (iron oxide nanoparticles) together with a mixture of solvent, free surfactant e.g. oleic acid, and other organic residues that are formed during the thermal decomposition reaction [34]. The workup procedure (see section 2) is a critical step to generate nanoparticle dispersions suitable for producing self-assembled arrays with a high quality and long-range order.

We have found that it is important to add a final step after the purification and workup where the nanoparticles dispersion is diluted with a few mL of toluene, then shaken for 1–2 h and finally gently vacuum treated at 40 °C to obtain a viscous, yet flowing, nanoparticle paste with a solid weight fraction of ≈40–50 wt%. The concentrated nanoparticle paste can be conveniently stored and diluted at later stages. We have observed that excessive purification/washing eventually leads to agglomeration of the nanoparticles, resulting in a poorly dispersable nanoparticle solid after vacuum drying. Due to oxidative degradation of the oleic acid coating, the concentrated nanoparticle paste should be stored in an inert atmosphere. Upon bench-top storage of the nanoparticle paste, there is a gradual loss of colloidal stability, resulting in inhomogeneous self-assembled arrays of poor quality. This is likely due to desorption of oleic acid from the particle surface over time or due to oxidation of the oleate ligands [43]. We have found that this type of ageing can be remedied by the addition of a small amount of fresh oleic acid, ca. 50 *μ*g of pure (99%) oleic acid per mg of iron oxide.

The decrease of the Néel relaxation frequency, in addition to the rapid scaling of dipolar interactions between the particles moments (the dipolar interaction scales: *U*_dd_ 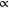 *l* ^6^ for two aligned nanocubes with edge length *l*) makes it impossible to obtain truly stable dispersions of the larger oleic acid coated nanocubes (C187).

**Figure 6.**
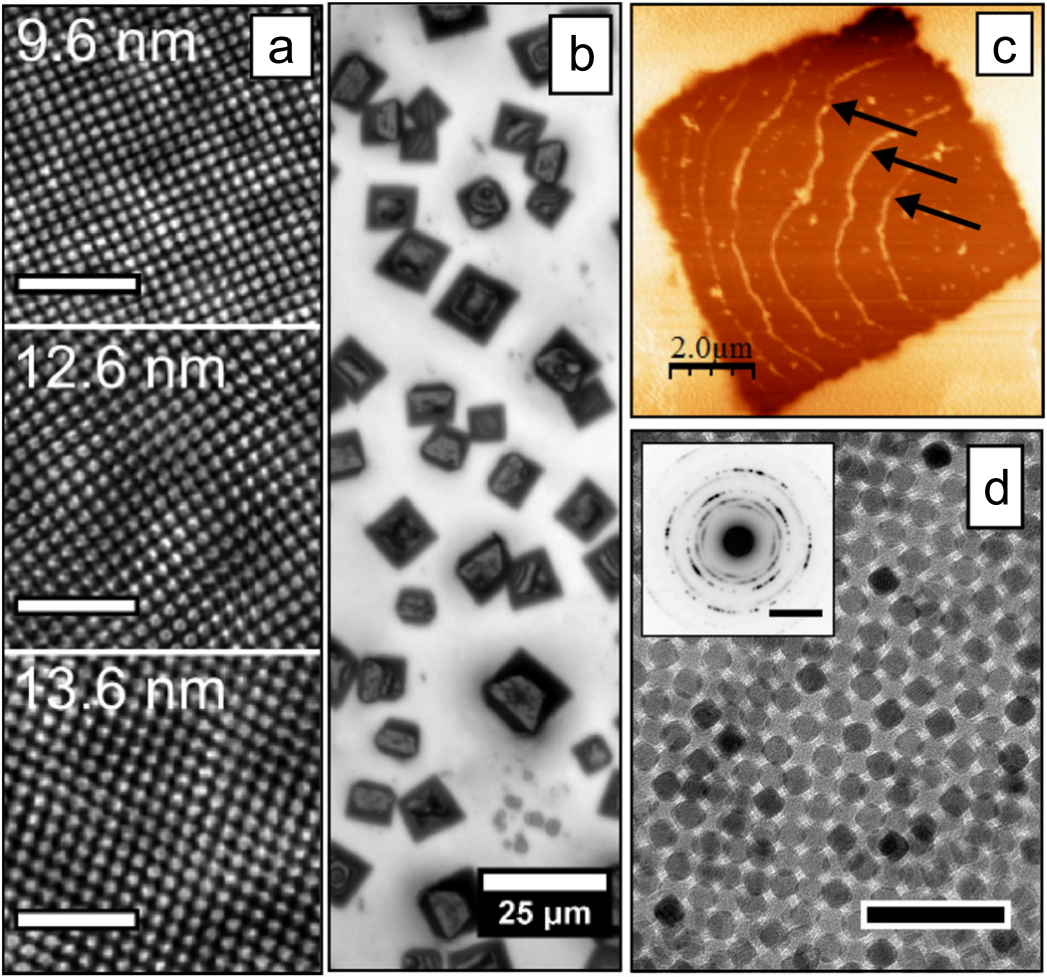
Self-assembled arrays and mesocrystals formed by the cubic nanoparticles described in this work. (a) High resolution scanning electron microscopy (SEM) images of ordered arrays of nanocubes taken from top-surfaces of self-assembled mesocrystals. Scale bars (white): 100 nm. The images have been FFT-filtered for clarity. (b) Reflected light microscopy images of cuboidal mesocrystals composed of 9.6 nm nanocubes by a conventional drop-casting procedure. (c) Atomic force microscope (AFM) tapping-mode phase image of the surface of a single cuboidal mesocrystal. Growth steps on the crystal surface are highlighted by arrows. (d) TEM image of a multilayer of 9.6 nm nanocubes. Scale bar (black): 50 nm. The inset shows a wide angle electron diffraction pattern of the area. Scale bar (inset): 5 nm^−1^.

Figure 6(a) shows that the monodisperse iron oxide nanocubes: C096, C126 and C136 (*σ*_std_ ≈ 5%), all form stable dispersions in toluene and self-assemble into large, ordered and crystallographically oriented arrays (i.e. mesocrystals [44, 45]) upon deposition and evaporation of the carrier solvent on various surfaces (e.g. Au, SiO_2_). In a typical drop-casting experiment (see section 2), mesocrystal growth occurs when most of the solvent has evaporated and the thickness of the remaining dispersion film is on the order of a few *μ*m [44]. Under controlled evaporation of the carrier solvent in a closed container it is possible to form mesocrystals with lateral dimensions of several tens of *μ*m. We have found that the height of the mesocrystals is locally uniform as a result of growth confinement within the thin dispersion film [44], and varies (typically between 500 nm and a few *μ*m) with the starting concentration of the nanoparticle dispersion (2–8 mg iron oxide/mL) .

Growth of iron oxide mesocrystals shares many aspects with classical crystal growth. The mesocrystals composed of 9.6 nm nanocubes shown in figure 6(b) are cuboidal as a result of their body-centered tetragonal (bct) mesostructure [44, 46]. It is also in some cases possible to observe step edges on the flat top surfaces of the mesocrystals (see figure 6(c)). Figure 6(d) shows that a self-assembled nanocube multilayer with a bct structure exhibits a somewhat smeared crystallographic texture. This is evident from the in-arc distribution of intensity in the selected area electron diffraction pattern (inset), resulting from the partial alignment of the nanocubes crystal axes.

## 4. Conclusions

We have demonstrated the synthesis of nanocubes and nanospheres over a broad size range by thermal decomposition of an iron(III) oleate precursor. We have demonstrated that the size of the nanoparticles can be tuned by changing the reaction temperature resulting in particles with very narrow size distributions. The use of high purity reagents in the synthesis and the addition of oleic acid or sodium oleate to the synthesis solution enable the particle shape to be tuned in a reproducible manner, from spheres when only oleic acid is added to cubes by addition of sodium oleate. Moreover, characterization of the nanoparticle shape by image analysis showed that the particles progressively became more cube-like as the amount of sodium oleate was increased. When using reagents of lower purity, the synthesis results were more erratic and the fine control over the absolute size and shape of the nanoparticles was lost. Finally, we have in detail described the purifications steps required to prepare dispersions that readily self-assemble under the evaporation of carrier solvent. Particles in the 10–14 nm range were assembled into ordered arrays, i.e. mesocrystals, with lateral dimensions of several tens of *μ*m.
